# High-Dose Melatonin and Ethanol Excipient Combined with Therapeutic Hypothermia in a Newborn Piglet Asphyxia Model

**DOI:** 10.1038/s41598-020-60858-x

**Published:** 2020-03-03

**Authors:** Nicola J. Robertson, Ingran Lingam, Christopher Meehan, Kathryn A. Martinello, Adnan Avdic-Belltheus, Liane Stein, Mohamed Tachrount, David Price, Magdalena Sokolska, Alan Bainbridge, Mariya Hristova, Bobbi Fleiss, Boris W. Kramer, Pierre Gressens, Xavier Golay

**Affiliations:** 1 University College London, London, WC1E 6HX UK; 2 University College London Hospitals NHS Trust, London, UK; 3 RMIT, Melbourne, Australia; 4 University of Maastricht, Maastricht, The Netherlands; 5 INSERM, Paris, France

**Keywords:** Neuroscience, Physiology

## Abstract

With the current practice of therapeutic hypothermia for neonatal encephalopathy, disability rates and the severity spectrum of cerebral palsy are reduced. Nevertheless, safe and effective adjunct therapies are needed to optimize outcomes. This study’s objective was to assess if 18 mg/kg melatonin given rapidly over 2 h at 1 h after hypoxia-ischemia with cooling from 1–13 h was safe, achieved therapeutic levels within 3 h and augmented hypothermic neuroprotection. Following hypoxia-ischemia, 20 newborn piglets were randomized to: (i) Cooling 1–13 h (HT; n = 6); (ii) HT+ 2.5% ethanol vehicle (HT+V; n = 7); (iii) HT + Melatonin (HT+M; n = 7). Intensive care was maintained for 48 h; aEEG was acquired throughout, brain MRS acquired at 24 and 48 h and cell death (TUNEL) evaluated at 48 h. There were no differences for insult severity. Core temperature was higher in HT group for first hour after HI. Comparing HT+M to HT, aEEG scores recovered more quickly by 19 h (p < 0.05); comparing HT+V to HT, aEEG recovered from 31 h (p < 0.05). Brain phosphocreatine/inorganic phosphate and NTP/exchangeable phosphate were higher at 48 h in HT+M versus HT (p = 0.036, p = 0.049 respectively). Including both 24 h and 48 h measurements, the rise in Lactate/N-acetyl aspartate was reduced in white (p = 0.030) and grey matter (p = 0.038) after HI. Reduced overall TUNEL positive cells were observed in HT+M (47.1 cells/mm^2^) compared to HT (123.8 cells/mm^2^) (p = 0.0003) and HT+V (97.5 cells/mm^2^) compared to HT (p = 0.012). Localized protection was seen in white matter for HT+M versus HT (p = 0.036) and internal capsule for HT+M compared to HT (p = 0.001) and HT+V versus HT (p = 0.006). Therapeutic melatonin levels (15–30mg/l) were achieved at 2 h and were neuroprotective following HI, but ethanol vehicle was partially protective.

## Introduction

Intrapartum-related neonatal encephalopathy (NE) is a major healthcare problem. Worldwide in 2010, NE accounted for 287,000 deaths and 400,000 survivors with impairment^1^. NE cannot be prevented in most cases and therapies are limited. The incidence of NE in Western Europe is 1–3/1000 term births and in low- and mid-resource settings the incidence is ~10 times higher^1,2^. Over the last 2 decades, in settings with neonatal intensive care facilities, therapeutic hypothermia (HT) is routinely used for moderate-to-severe NE, improving survival and reducing disability^3^. However, although the severity of cerebral palsy has reduced with HT^4^, survivors have significantly lower cognitive scores which are on average 14 IQ points lower than matched peers even in the absence of cerebral palsy at school-age^5^. Further adjustments to HT protocols do not improve outcome^6,7^, therefore adjunct therapies to augment HT protection are urgently needed.

Pre-clinical studies suggest that melatonin (N-acetyl-5-methoxytryptamine) in pharmacologic levels is safe and neuroprotective for hypoxic-ischemic injury in the adult^8^ and neonatal^9^ brain, mediated by anti-oxidant, anti-apoptotic and anti-inflammatory properties^10,11^. Extrapolating from *in vitro*^12^ and pre-clinical piglet studies^13,14^ showing melatonin reduces cell death in a concentration-dependent manner, we deduce that a plasma level 15–30 mg/l is required for optimal protection. As melatonin is sparingly soluble, solubility enhancers, such as ethanol, are used. Ethanol could confound previous studies of melatonin protection^13,15–19^, as low-dose ethanol ~4 h after HI is protective in adult models of stroke^20–22^. In a previous piglet study, we observed augmentation of HT protection with 30 mg/kg/24 h melatonin with ethanol excipient when started 10 mins after hypoxia-ischemia (HI), reaching blood levels >15 mg/l within 1 h^13^. In a subsequent study using a proprietary melatonin formulation (15 mg/kg), without ethanol excipient, given 2–8 h after HI, there was less clear protection with therapeutic levels reached only at 8 h^14^. In this same study, no protection was seen with 5 mg/kg melatonin started at 2 h (blood melatonin <4 mg/l)^14^. Taken together, these studies suggest melatonin protection is dose-dependent, time critical and influenced by excipient. With the aim to achieve therapeutic levels within 3 h of HI, we subsequently performed pharmacokinetic (PK) modelling, suggesting optimised melatonin dosing would be 18 mg/kg/24 h over 2 h started 1 h after HI^14^.

We used a piglet model with similar protocols to our neonatal intensive care unit and similar sequences for magnetic resonance spectroscopy (MRS) as NE babies. Our objective was to assess safety, efficacy and PK of an optimized melatonin-dosing regimen based on prior PK-modelling. We hypothesized that 18 mg/kg melatonin started 1 h after HI, infused over 2 h would reach therapeutic levels within 3 h and augment HT. To clarify any influence of ethanol vehicle on neuroprotection, we studied ethanol with and without melatonin. Primary outcome measures were: (i) Cerebral MRS biomarkers (proton (1H) and phosphorus (^31^P) MRS). Thalamic lactate/N-acetyl aspartate (Lac/NAA) is the most accurate outcome biomarker at 2-years^23,24^, used in clinical neuroprotection trials^25^, with clear superiority over other MR methods^24^. ^31^P MRS is less accessible on MRI systems but has defined secondary energy failure in NE^26^ and its relation with 1-year brain growth and outcome^27^; (ii) aEEG recovery; aEEG is used in NE babies during cooling and recovery predicts outcome^28^; (iii) Quantitative cell death in 8 brain regions (TUNEL-positive cells) at 48 h.

## Materials and Methods

### Animal experiments, surgical preparation and randomization

**Figure 1:**
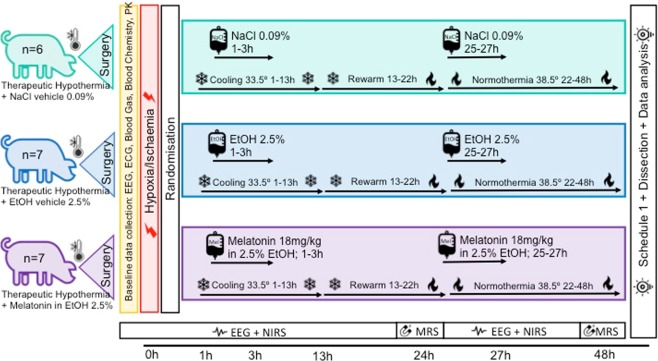
Study time-line. Following baseline data acquisition, piglets underwent cerebral HI. At the end of HI (time t = 0), piglets were randomized to (i) HT (33.5 °C) from 1–13 h with saline bolus at 1–3 and 25–27 h or (ii) HT+vehicle (HT+V; 2.5% ethanol); (iii) HT+melatonin (HT+M;18 mg/kg dissolved in 2.5% ethanol) at 1–3 and 25–27 h. Intensive care support was given for 48 h following HI. aEEG/continuous video EEG was acquired. MRI/MRS was performed at 3 T at 24 and 48 h. The experiment was terminated at 48 h and brain immunohistochemistry analyzed.

All animal experiments were approved by the UCL Ethics Committee and performed according to UK Home Office Guidelines [Animals (Scientific Procedures) Act, 1986]. The study complies with ARRIVE guidelines. Large White male piglets were anesthetized, surgically prepared and intensive care maintained as described previously^29^. Criteria for study entry were: (i) normal aEEG/EEG at baseline after surgery; (ii) no pyrexia; (iii) no aEEG recovery within 1 h of HI. The experimental plan is shown in Fig. 1.

Following HI and resuscitation, animals were randomized to (i) Therapeutic hypothermia 1–13 h (HT) (0.9% saline (7.2 ml/kg/h) at 1–3 h and 25–27 h); (ii) HT+Vehicle (HT+V) (2.5% ethanol (7.2 ml/kg/h) at 1–3 h and 25–27 h); (iii) HT+ Melatonin (HT+M) (18 mg/kg (7.2 ml/kg/h) at 1–3 h and 25–27 h). Intensive care support for the animal throughout 48 h and complications were managed in accordance with local neonatal intensive care guidelines.

### Transient cerebral hypoxia ischemia

Compared to the original studies based on assessment of NTP reduction during HI on ^31^P MRS^29^, the monitoring and titration of the HI insult were modified recently in this and other studies^30^. Baseline physiological observations and aEEG were monitored prior to HI. Carotid occluders were inflated and fraction of inspired oxygen (FiO_2_) reduced simultaneously at the start of the insult. FiO_2_ was decreased to 6% over the first 3 min and titrated to mean blood pressure and EEG. Oxygen delivery was increased in the event of a mean BP <27 mmHg and restricted further if recovery of EEG activity was observed during the insult. Blood gas analysis was performed at 5 min intervals during HI. Total duration of HI was anticipated to be 20–25 min, depending on the duration of isoelectric EEG, hypotension (mean BP <30 and <25 mmhg), total reduction in FiO_2_ (AUC FiO_2_) and severity of acidosis on blood gas analysis. At the end of the insult, the animal was resuscitated, occluders deflated and FiO_2_ increased to air.

### Melatonin administration

#### Melatonin preparation and delivery

Melatonin (Sigma-Aldrich) was dissolved in ethanol and 0.9% NaCl (2.5% v/v vehicle) in the dark shortly before administration. 18 mg/kg melatonin (7.2 ml/kg/h) was infused intravenously over 2 h starting at 1 h after HI and at 25 h after HI. Blood was sampled at baseline and 2, 3, 6, 24, 26, 27, 36, 42, 48 h after time 0 (end of HI).

### Magnetic resonance spectroscopy

**Figure 2:**
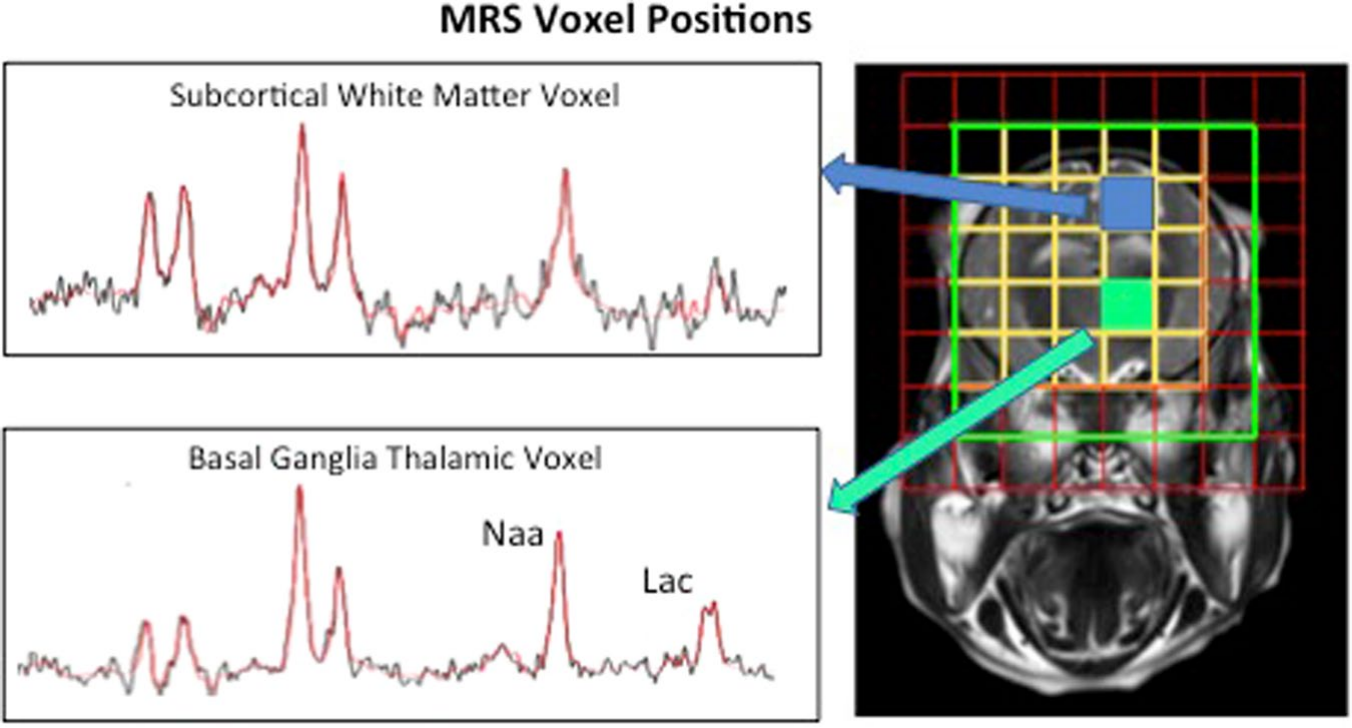
Representative spectra and voxel positions for ^1^H MRS. Spectroscopy data were collected from voxels located in the dorsal right subcortical white matter at the centrum semiovale level and from two voxels in the deep grey matter centred on both thalami.

^31^P and ^1^H MRS was performed at 24 and 48 h after HI in a Philips clinical 3 T MRI scanner. ^31^P metabolites were measured over whole brain (see supplementary file). ^1^H MRS metabolites were measured in white matter in the right subcortical region (8 × 8 × 15 mm) and deep grey matter (15 × 15 × 10 mm) in the thalamus **(**Fig. 2**)**. Data was analyzed using jMRUI and Lac/NAA peak area ratio calculated.

### Amplitude integrated electroencephalogram (aEEG)

A multichannel EEG and aEEG (Nicolet) was acquired at baseline and continued for 48 h post-insult. The aEEG score was based on pattern classification^31^; isoelectric (0), continuous low voltage (1), burst suppression (2), discontinuous normal voltage (3) and continuous normal voltage (4).

### Immunohistochemistry

At 48 h after HI, piglets were euthanized with pentobarbital and the brain fixed by cardiac perfusion with cold 4% paraformaldehyde, dissected out and post-fixed at 4 °C in 2% paraformaldehyde for 7 days. Coronal slices (5 mm thick) of the right hemisphere, starting from anterior to the optic chiasma, were embedded in paraffin, sectioned to 8 µm thickness and stained with hematoxylin and eosin to validate the bregma for analysis. For each animal, 2 sections (bregma 00 and −2.0) were stained and 8 brain regions were examined: cingulate cortex, sensorimotor cortex, hippocampus, internal capsule, periventricular white matter, caudate, putamen and thalamus. Histological analysis was performed by an investigator blinded to treatment allocation^32^.

Please see Supplementary file for details on immunohistochemistry methodology and statistical analysis.

## Results

One piglet (HT+M) died at 21 h and one piglet (HT) was excluded due to aEEG recovery within 1 h of HI, suggesting mild HI. Twenty piglets were included; HT (n = 6), HT+V (n = 7); HT+M (n = 7).

### Physiological data and insult severity

There were no intergroup differences for bodyweight, baseline heart rate, MABP and core temperature **(**Table 1**)**. The arterial gases at baseline were similar. Considering HI severity, the duration of (i) hypoxia and carotid occlusion, (ii) isoelectric EEG and (iii) MABP <25 and <30 mmHg were similar **(**Table 1**)**. At the end of HI, blood pH, lactate, base excess and AUC FiO_2_ reduction below 21% were similar **(**Table 1**)**.

**Table 1:** Physiological parameters for the piglets in each group.

Parameter	HT LS Mean (SEM)	HT+Vehicle LS Mean (SEM)	HT+Melatonin LS Mean (SEM)	P value
Weight (kg)	2.1 (0.05)	2.1 (0.04)	2.0 (0.05)	0.776
**Hypoxic ischemic insult**				
Duration of HI insult (min)	21.3 (1.1)	20.0 (1.0)	23.1 (1.0)	0.103
Duration of isoelectric EEG during HI (min)	19 (0.08)	18.3 (0.8)	20.9 (0.8)	0.081
Duration of blood pressure <30 mmHg (min)	8.7 (1.4)	10.7 (1.3)	6.9 (1.3)	0.150
Duration of blood pressure <25 mmHg (min)	2.3 (1.2)	3.2 (1.1)	0.7 (1.1.)	0.321
Nadir pH	7.2 (0.03)	7.2 (0.03)	7.2 (0.03)	0.433
Nadir Lactate (mmol/l)	14.3 (0.7)	11.9 (0.7)	13.3 (0.7)	0.070
Area under the curve FiO_2_	287.2 (19.6)	271.6 (18.2)	322.4 (18.2)	0.160
Nadir base excess (mmol/l)	−13.2 (2.94)	−9.7 (1.2)	−9.6 (1.2)	0.103
**Heart rate (min**^**−1**^**)**				
Baseline	179.9 (8.2)	166.2 (7.6)	175.4 (7.6)	0.471
0–1 h after insult	200 (10.0)	197.6 (9.2)	192.6 (9.2)	0.857
1–13 h	181.8 (6.82)	171.3 (6.3)	171.0 (6.3)	0.445
13–25 h	202 (11.5)	195.6 (10.6)	181.4 (10.6)	0.416
25–48 h	212.4 (9.4)	182.1 (7.9)	187.1 (7.9)	0.061
**Mean arterial blood pressure (mmHg)**				
Baseline	52 (2.6)	51.2 (2.4)	50.2 (2.4)	0.875
0–1 h after insult	53.3 (5.0)	44.3 (4.6)	45.4 (4.6)	0.384
1–13 h	46 (1.6)	42.4 (1.5)	45.8 (1.5)	0.191
13–25 h	45.8 (2.5)	42.6 (2.3)	50.3 (2.3)	0.091
25–48 h	55.0 (2.1)	49.4 (1.8)	52.6 (1.8)	0.152
**Rectal temperature (°C)**				
Baseline	38.1 (0.3)	38.3 (0.3)	38.0 (0.3)	0.777
**0–1 h after insult**	**38.9 (0.2)**	**38.5 (0.1)**	**38.2 (0.1)**	**0.020**
1–13 h	34.0 (0.05)	34 (0.05)	33.9 (0.05)	0.615
13–25 h	36.2 (0.1)	36.5 (0.1)	36.4 (0.1)	0.295
25–48 h	38.4 (0.09)	38.5 (0.08)	38.4 (0.08)	0.794
**PaO**_**2**_ **(kPa)**				
Baseline	11.1 (1.3)	9.9 (1.2)	14.2 (1.2)	0.067
End of insult (time 0)	9.3 (1.2)	6.4 (1.1)	7.6 (1.1)	0.241
12 h after time 0	11.5 (1.0)	11.3 (0.9)	10.8 (0.9)	0.879
24 h after time 0	11.5 (1.0)	11.4 (0.9)	12.8 (0.9)	0.469
48 h after time 0	14.1 (0.7)	12.0 (0.6)	12.7 (0.6)	0.119
**PaCO**_**2**_ **(kPa)**				
Baseline	5.6 (0.4)	6 (0.4)	5.4 (0.4)	0.506
End of insult (time 0)	5.6 (0.4)	6.1 (0.4)	6 (0.4)	0.628
12 h after time 0	4.8 (0.5)	5.4 (0.5)	5.6 (0.5)	0.521
**24 h after time 0**	**7 (0.3)**	**5.6 (0.3)**	**5.5 (0.3)**	**0.003**
48 h after time 0	5 (0.3)	4.5 (0.3)	5.2 (0.3)	0.311
**Blood pH**				
pH_Baseline	7.4 (0.03)	7.4 (0.03)	7.5 (0.03)	0.301
End of insult (time 0)	7.2 (0.04)	7.2 (0.03)	7.2 (0.03)	0.960
12 h after time 0	7.5 (0.04)	7.5 (0.03)	7.5 (0.03)	0.641
**24 h after time 0**	**7.3 (0.03)**	**7.4 (0.02)**	**7.5 (0.02)**	**0.009**
48 h after time 0	7.5 (0.02)	7.5 (0.02)	7.5 (0.02)	0.743
**Base excess (mmol/l)**				
Baseline	1.8 (1.7)	3.3 (1.6)	5.4 (1.6)	0.319
End of insult (time 0)	−10.0 (1.7)	−9.7 (1.6)	−9.6 (1.6)	0.982
12 h after time 0	5.7 (1.5)	4.7 (1.4)	5.7 (1.4)	0.845
24 h after time 0	2.2 (2.6)	−0.4 (2.2)	5.1 (2.2)	0.222
48 h after time 0	2.8 (1.3)	1.9 (1.1)	3.1 (1.1)	0.689
**Lactate (mmol/l)**				
Baseline	5.2 (0.7)	3.8 (0.7)	4.1 (0.7)	0.347
End of insult (time 0)	12.9 (1.0)	12.3 (0.9)	13.3 (0.9)	0.756
12 h after time 0	3.1 (0.6)	3.9 (0.6)	3.1 (0.6)	0.543
24 h after time 0	3.3 (1.5)	5.3 (1.3)	2.1 (1.3)	0.227
48 h after time 0	1.2 (0.3)	1.7 (0.2)	1.5 (0.2)	0.425
**Glucose (mmol/l)**				
Baseline	6.4 (0.5)	6 (0.5)	5.6 (0.5)	0.493
End of insult (time 0)	9.3 (1.1)	9.2 (1.0)	9.7 (1.2)	0.942
12 h after time 0	15.2 (1.6)	13.3 (1.5)	11.3 (1.5)	0.254
24 h after time 0	17 (3.9)	13.2 (3.3)	8.9 (3.3)	0.307
48 h after time 0	7.2 (1.0)	6.4 (0.9)	6 (0.9)	0.657

Time zero = time of resuscitation after HI. Least square mean values (SEM) are presented for the three groups: (i) HT (n = 6), (ii) HT+Vehicle(Ethanol) (n = 7), and (iii) HT+Melatonin (n = 7). An analysis of variance (ANOVA) model was fitted to each group at each time point. No differences were observed between any groups at any time point or ranges following Bonferonni multiplicity correction (p < 0.001). Bold figures represent those measurements that are significantly different between groups.

In the first hour after HI, the mean core temperature was higher in HI versus HI+M (38.9 °C versus 38.2 °C, p = 0.006), although temperatures were within 0.4 °C of normal piglet temperature (38.5 °C). For the remainder of the study, there was no temperature difference. The blood pH was more alkalotic and PaCO_2_ lower at 24 h after HI in HT+M (p = 0.001) and HT+V (p = 0.003) versus HT.

There was no difference in saline boluses. Inotropic use with Dopamine and Dobutamine was higher in HT and HT+V versus HT+M. Noradrenaline and adrenaline use did not differ **(**Table 2**)**.

**Table 2:** Average total volume replacement and inotrope infusion for the piglets in the HT, HT+V, HT+M groups during the 48 h after HI.

Infusions	HT		HT+V		HT+M		P value
	Mean (SD)		Mean (SD)		Mean (SD)		
Dopamine (µg/kg/min)	15.3	4.5	16.7	3.5	8.9	4.1	0.005
Dobutamine (µg/kg/min)	2.7	5.4	7.1	6.4	0.7	1.9	0.07
Noradrenaline (ng/kg/min)	13.0	23.2	43.5	70.3	17.9	47.4	0.53
Adrenaline (ng/kg/min)	157.0	348.5	192.4	477.9	14.2	37.6	0.60
10 ml/kg Saline Bolus (n)	0.2	0.4	0.4	1.1	0.1	0.4	0.73

### aEEG recovery and seizures

**Figure 3:**
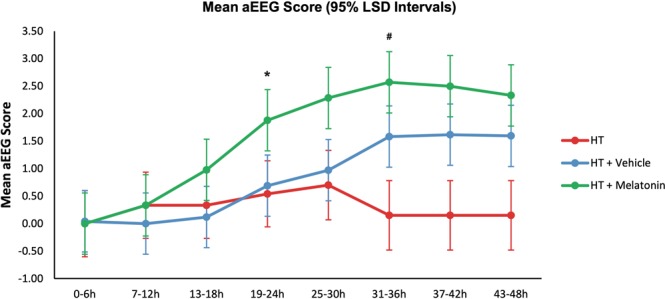
Amplitude-integrated electroencephalogram (aEEG) background activity at baseline, during HI and following HI. The grouped mean hourly aEEG scores per 6 h period with 95% Least Significant Difference (LSD) are shown. Non-overlapping bars show evidence of a significant difference. The mean hourly aEEG scores were significantly higher in the HT+M group versus HT+V and HT from 19–24 h (p = 0.037 and p = 0.025 respectively) and 25–30 h (p = 0.022 and p = 0.010 respectively) after HI, indicating faster recovery of brain electrical activity. From 31 h onwards, the vehicle treated group’s brain electrical activity recovered and there was no difference between the HT+M and HT+V groups. The aEEG background voltage did not recover in the HT group and was significantly lower than both HT+V and HT+M groups, with scores of <1 from 31 h until the end of the study. *Shows when the HT+M group aEEG recovered compared to HT+V and HT groups. ^#^Shows when the HT+V group aEEG recovered compared to the HT group.

aEEG background activity scores were 4 (normal) in all piglets before HI and 0 during HI. Following HI, the mean hourly aEEG scores were higher in HT+M versus HT+V and HT from 19–24 h (p = 0.037 and p = 0.025 respectively) and 25–30 h (p = 0.022 and p = 0.010) after HI, indicating faster recovery of electrical activity with melatonin-augmented cooling. From 31 h, the vehicle treated group’s brain electrical activity recovered and there was no difference between HT+M and HT+V. aEEG background voltage did not recover in HT and was lower than both HT+V and HT+M, with scores of <1 from 31 h until the study end (Fig. 3).

One piglet in HT and 3 piglets each in HT+V and HT+M had seizures. All electrographic seizures were treated with 20 mg/kg phenobarbitone; short clinical seizures with no electrographic evidence of seizures were not treated. One animal in HT and HT+M and 3 animals in HT+V received phenobarbitone.

### 3T MRS

**Figure 4:**
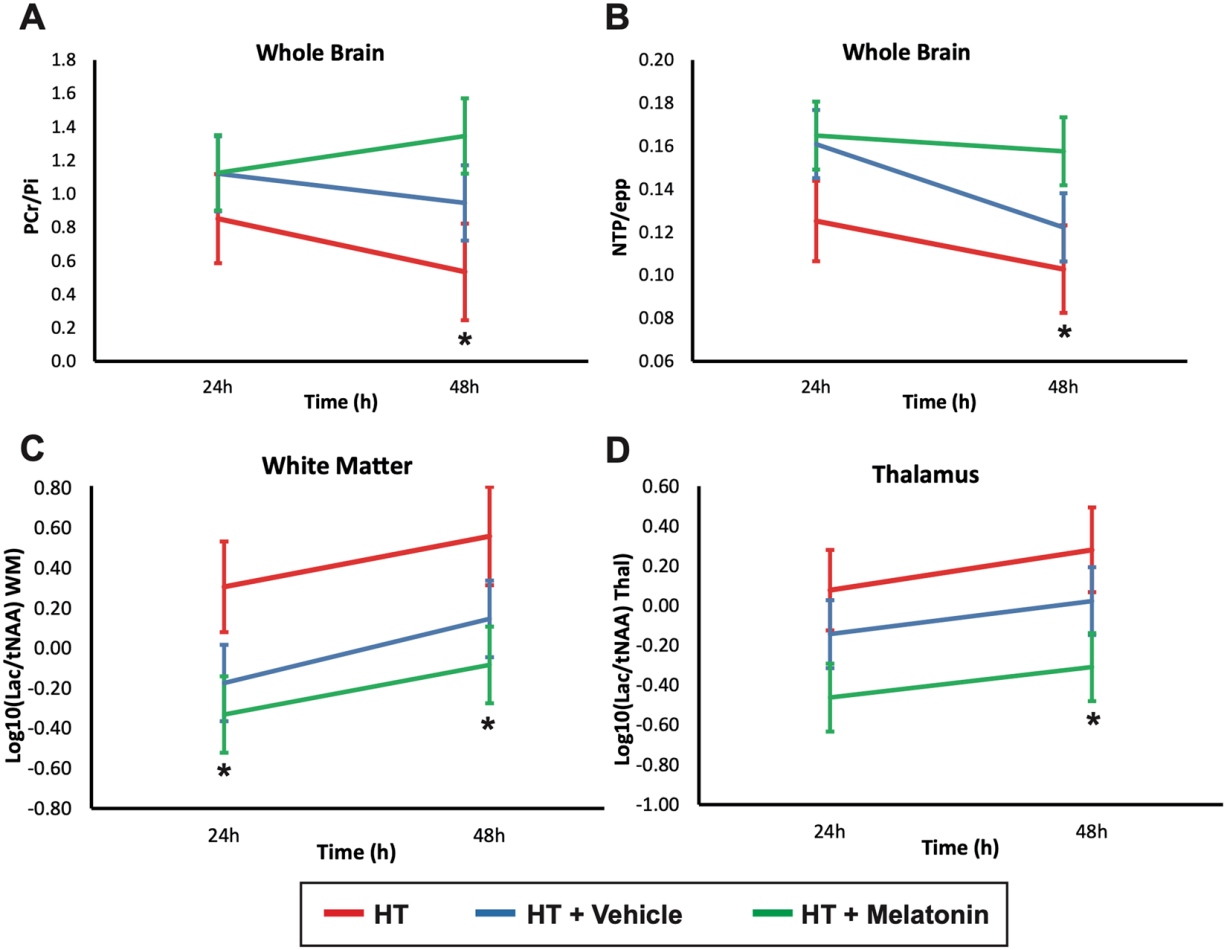
1H Magnetic resonance spectroscopy of the brain at 24 and 48 h after HI. Least square mean plots with 95% Least Significant Difference (LSD) bars for the NTP/epp and PCr/Pi in whole-forebrain, and Lac/NAA in thalamus and white matter; non-overlapping bars show evidence of a significant difference. NTP/epp (**A**) and PCr/Pi (**B**) means were significantly higher in the HT+M group compared to HT at 48 h (p = 0.049 and 0.036 respectively). Comparing the HT+M group to the HT group including both 24 and 48 h, Lac/NAA was lower in the white matter (p = 0.30) and grey matter (p = 0.038). There were no significant differences in MRS measures comparing HT and HT+V groups and HT+V and HT+M groups. epp = exchangeable phosphate pool; Lac = lactate; NAA = N-acetyl aspartate; Thal = thalamus; WM = white matter; HI = hypoxia ischemia; M = melatonin *p < 0.05.

48 h after HI, comparing HT+M to HT, there was higher PCr/Pi (p = 0.036) and NTP/epp (p = 0.049). Comparing HT+M to HT including 24 and 48 h data, Lac/NAA was lower in the white matter (p = 0.30) and grey matter (p = 0.038). There were no differences in MRS comparing HT and HT+V or HT+V and HT+M (Fig. 4).

### Immunohistochemistry

#### TUNEL

**Figure 5:**
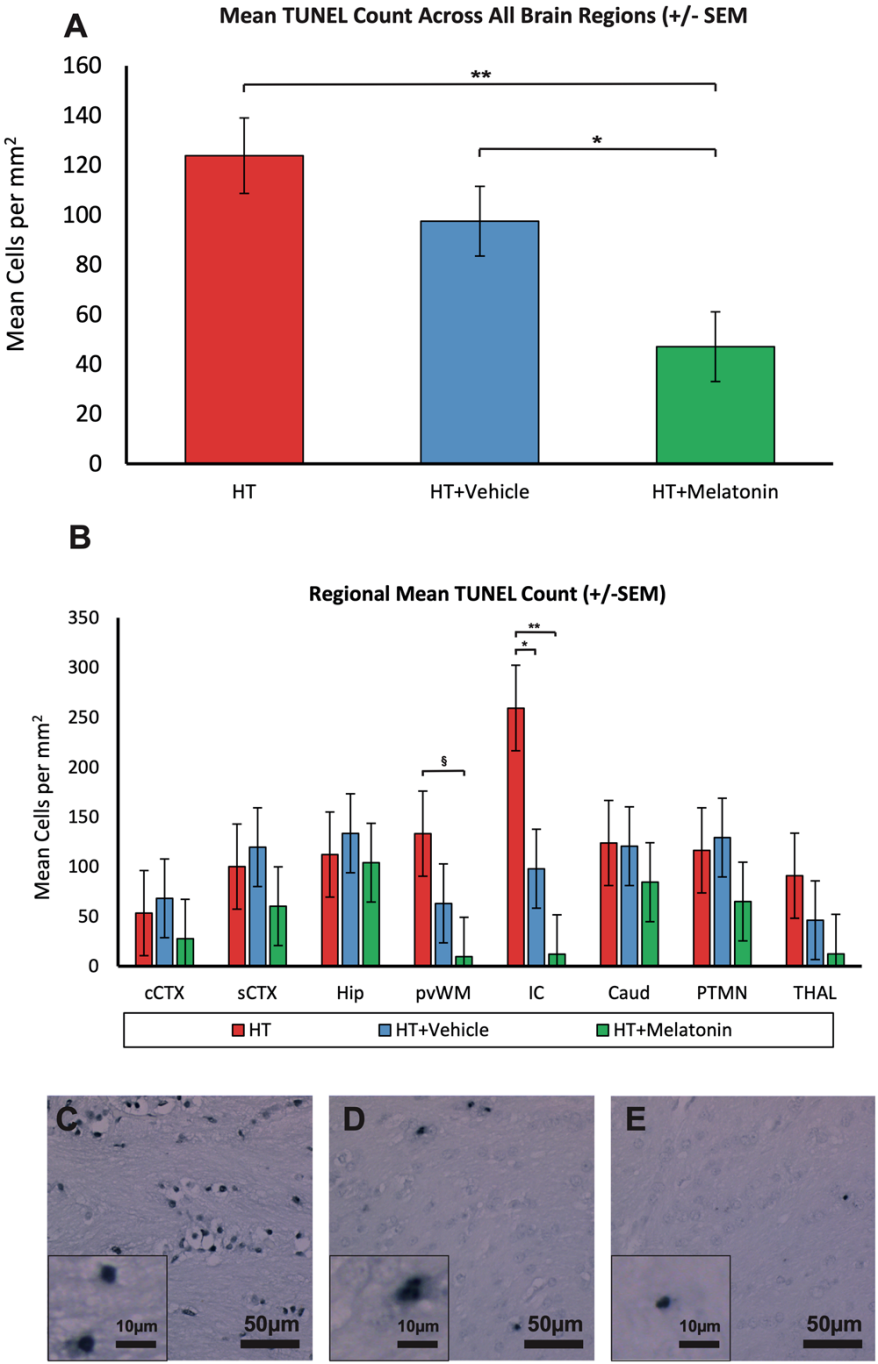
(**A**) TUNEL immunohistochemistry, all brain regions. Co-treatment of cooling with melatonin decreased overall TUNEL positive cell death at 48 h after HI when compared to cooling and vehicle (p = 0.012) and cooling alone (p = 0.003). *p = 0.012 HT+M versus HT+V. **p = 0.003 HT+M versus HT. (**B**) TUNEL immunohistochemistry from 8 brain regions. There were less TUNEL-positive cells in the IC in HT+M compared to both HT+V and HT groups. There were fewer TUNEL-positive cells in the pvWM in the HT+M group versus HT alone. **p < 0.001 HT+M versus HT. *p < 0.01 HT+V versus HT. ^§^p > 0.05 HT+M versus HT. Internal capsule = IC; pvWM = periventricular white matter. Error bars represent standard error.

TUNEL-positive cells/mm^2^ for treatment groups are shown in Fig. 5 with group comparison in Table 3. Over all regions, TUNEL-positive cells/mm^2^ were 123.8 cells/mm^2^ in HT, 97.5 cells/mm^2^ in HT+V and 47.1 cells/mm^2^ in HT+M groups. There were lower TUNEL-positive cells comparing HT+M versus HT+V (p = 0.024) and HT+M versus HT (p = 0.001). For regional differences; there were lower TUNEL-positive cells in the IC in HT+M (12.2 cells/mm^2^) versus HT (259.6 cells/mm^2^; p < 0.001). In the same region, there was a partial protective effect with ethanol with lower TUNEL-positive cells in HT+V (98.1 cells/mm^2^) versus HT (259.6 cells/mm^2^; p = 0.006). In the pvWM there were less TUNEL-positive cells in HT+M (9.7 cells/mm^2^) versus HT (133.4cells/mm^2^; p = 0.036).

**Table 3:** Least Square mean (SEM) TUNEL positive cells (per mm^2^) in each brain region and overall.

TUNEL positive cells per mm^2^ Least square mean (SEM)					
	HT	HT+V	HT+M	P values for difference in means	
cCTX	53.4 (42.8)	68.3 (39.6)	27.7 (39.6)	HT v HT+M	p = 0.47
				HT v HT+V	p = 0.80
				HT+V v HT+M	p = 0.66
sCTX	100.2 (42.8)	119.7 (39.6)	60.4 (39.6)	HT v HT+M	p = 0.50
				HT v HT+V	p = 0.74
				HT+V v HT+M	p = 0.29
Hip	112.4 (42.8)	133.7 (39.6)	104.3 (39.6)	HT v HT+M	p = 0.89
				HT v HT+V	p = 0.72
				HT+V v HT+M	p = 0.60
pvWM	133.4 (42.8)	63.3 (39.6)	9.7 (39.6)	**HT v HT+M**	p = 0.036
				HT v HT+V	p = 0.23
				HT+V v HT+M	p = 0.34
IC	259.6 (42.8)	98.1 (39.6)	12.2 (39.6)	**HT v HT+M**	p = 0.001
				**HT v HT+V**	p = 0.006
				HT+V v HT+M	p = 0.13
Caudate	124.1 (42.8)	120.8 (39.6)	84.6 (39.6)	HT v HT+M	p = 0.50
				HT v HT+V	p = 0.96
				HT+V v HT+M	p = 0.52
Putamen	116.6 (42.8)	129.4 (39.6)	65.1 (39.6)	HT v HT+M	p = 0.38
				HT v HT+V	p = 0.83
				HT+V v HT+M	p = 0.25
Thalamus	91.0 (42.8)	46.4 (30.6)	12.6 (39.6)	HT v HT+M	p = 0.18
				HT v HT+V	p = 0.45
				HT+V v HT+M	p = 0.55
Overall	**123.8 (15)**	**97.5 (14)**	**47.1 (14)**	**HT v HT+M**	p = 0.0003
				HT v HT+V	p = 0.200
				**HT+V v HT+M**	p = 0.012

P values for the difference in means are shown in the right hand column; significant values are shown in bold. SEM = standard error of the mean; cCTX = cingulate cortex; sCTX = sensorimotor cortex; pvWM = periventricular white matter; IC = internal capsule.

#### Iba1

**Figure 6:**
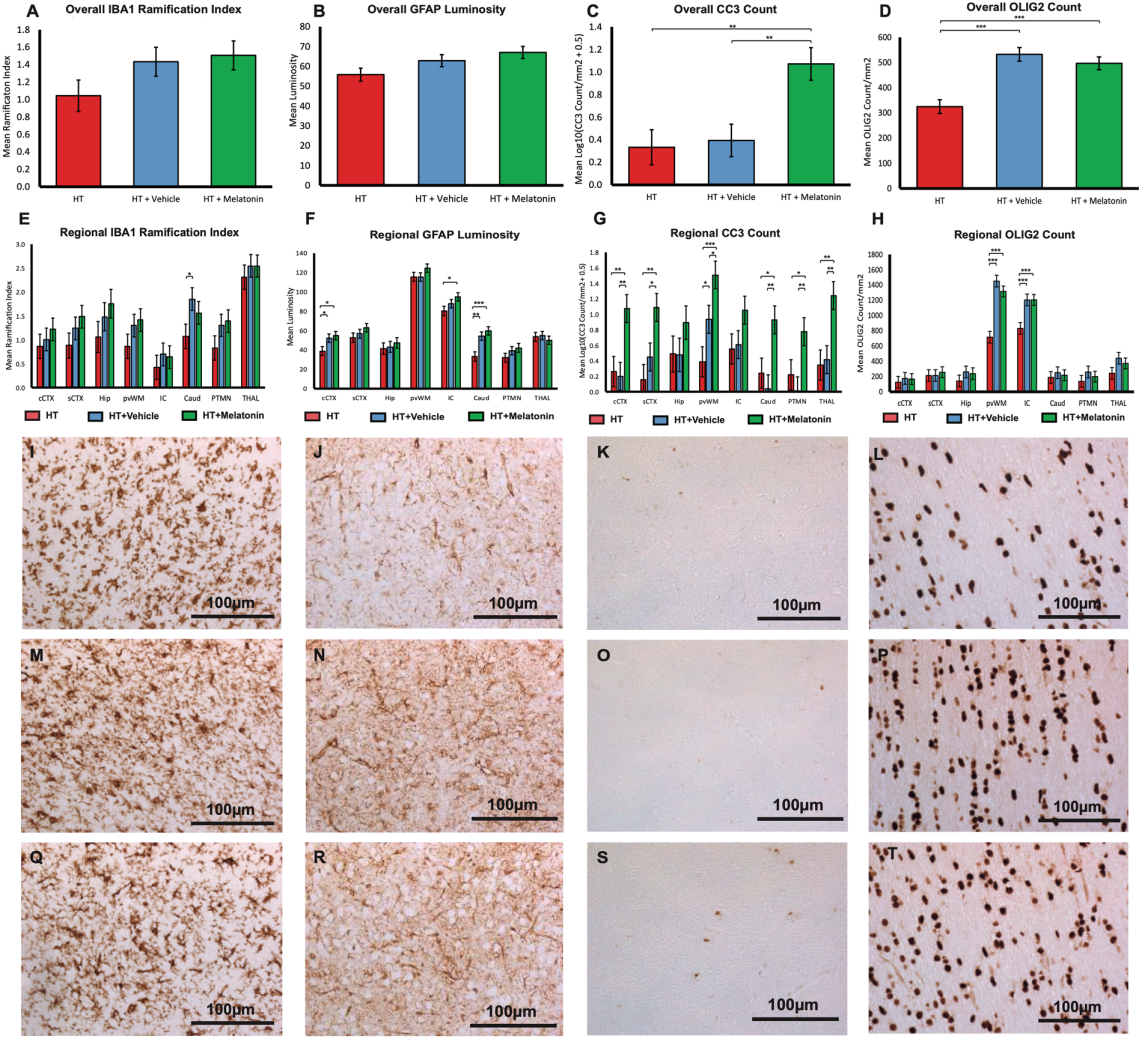
Overall and regional immunohistochemistry for HT, HT+V and HT+M groups for IBA1 ramification index, GFAP luminosity, Cleaved Caspase 3 (CC3) and OLIG2. For IBA1 ramification index, there was no difference in overall ramification index in the HT+M versus HT+V or HT alone (**A**). On regional analysis the caudate showed an increase in IBA1 ramification index in the HT+V versus HT (**E**). Representative photomicrographs are shown for HT (**I**), HT+V (**M**) and HT+M (**Q**). There was no difference in overall astrogliosis (GFAP) (**B**), but localized increases in astrogliosis in the cCTX, IC and caudate as shown **(F**). Representative GFAP photomicrographs are shown for HT (**J**), HT+V (**M**) and HT+M (**R**). There was an overall increase in CC3 in the HT+M versus HT and HT+M versus HT+V (**C**). Localized differences with increased CC3 mainly in the HT+M group were compared to both HT+V and HT alone were seen in cCTX, sCTX, pvWM, caudate, putamen, thalamus (**G**). Representative CC3 photomicrographs are shown for HT (**K**), HT+V (**O**) and HT+M (**S**). There was an overall increase in OLIG2 counts in the HT+M and HT+V compared to HT (**D**). Localised differences were seen with increased OLIG2 mainly in the IC and pvWM (**H**). Representative OLIG2 photomicrographs are shown for HT (**L**), HT+V (**P**) and HT+M (**T**). Cingulate cortex = cCTX; Sensorimotor cortex = sCTX; Hippocampus = HIP; Periventricular white matter = PvWM; Internal capsule = IC; Caudate = CAUD; Putamen = PTMN; Thalamus = THAL. Error bars represent standard error ***p < 0.001, **p < 0.01, *p < 0.05.

There was no difference in overall ramification index (higher index suggests less activation) in HT+M versus HT+V or HT **(**Fig. 6A**)**. On regional analysis the caudate showed increased IBA1 ramification index in HT+V versus HT **(**Fig. 6E**)**.

#### GFAP

On GFAP luminosity, there was no difference in overall astrogliosis **(**Fig. 6B**)**, but localized increases in cCTX, IC and caudate **(**Fig. 6F**)**.

#### Cleaved caspase 3 (CC3)

There was an overall increase in CC3 in HT+M versus HT and HT+M versus HT+V **(**Fig. 6C**)**. Localized differences with increased CC3 mainly in HT+M versus HT+V and HT were seen in cCTX, sCTX, pvWM, caudate, putamen, thalamus **(**Fig. 6G**)**.

#### OLIG2

The estimated mean OLIG2 cells/mm^2^ for are shown in Fig. 6D. Over all brain regions, OLIG2 cells/mm^2^ were 324.8 cells/mm^2^ in HT, 532.0 cells/mm^2^ in HT+V and 496.5 cell/mm^2^ in HT+M. There were higher numbers of OLIG2 cells in HT+V and HT+M versus HT (both p < 0.0001). For regional differences, there were higher OLIG2 counts in internal capsule in HT+V (1204.5 cells/mm^2^) versus HT (831.2 cells/mm^2^; p < 0.0001) and HT+M (1204.9 cells/mm^2^) versus HT (831.2 cells/mm^2^; p < 0.0001) **(**Fig. 6H**)**. There were higher OLIG2 counts in the pvWM in HT+V (1451.6 cells/mm^2^) versus HT (716.3 cells/mm^2^; p < 0.0001) and HT+M (1316.4 cells/mm^2^) versus HT (831.2 cells/mm^2^; p < 0.0001). Excluding IC and pvWM regions (variability was larger than other regions), we observed higher OLIG2 counts in: hippocampus, HT+V (260.9 cells/mm^2^) versus HT (142.3 cells/mm^2^; p = 0.041); putamen, HT+V (259.7cells/mm^2^) versus HT (137.6 cells/mm^2^; p = 0.036) and thalamus, HT+V (441.1 cells/mm^2^) versus HT (242.0 cells/mm^2^; p = 0.001) and HT+M (372.3 cells/mm^2^) versus HT (242.0 cellsmm^2^; p = 0.02).

### Pharmacokinetics

**Figure 7:**
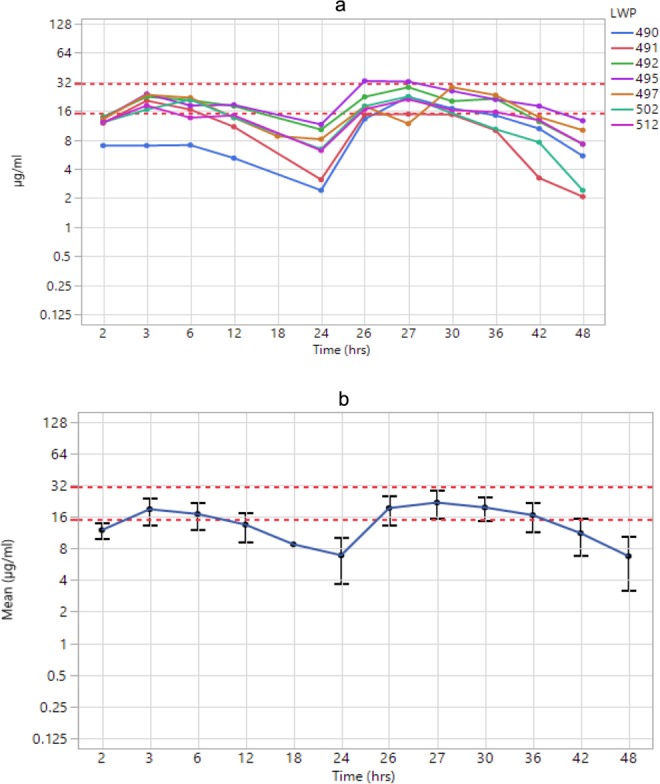
Melatonin plasma levels for individual piglets (**A**) and Mean (95% CI) Melatonin levels for the HT+M group (**B**). Melatonin reached the target therapeutic range at 2 h and 26 h after HI (1 h after each infusion started) and remained within this range for up to 18 h.

18 mg/kg melatonin infusion over 2 h starting at 1 h after HI led to plasma target therapeutic levels of melatonin at 2 h after HI. The peak melatonin level in the first 24 h was at 3 h after HI (18.84 µg/ml) and in the 2^nd^ 24 h at 27 h after HI (21.84 µg/ml). Individual subject profiles are in Fig. 7A and mean group plasma levels with 95% CI are in Fig. 7B.

## Discussion

Compared to therapeutic hypothermia alone, we observed improved cerebral protection with the addition of 18 mg/kg melatonin given intravenously at 1 h and 25 h after HI, based on aEEG recovery from 19 h, improved cerebral energy metabolism at 48 h on ^31^P and ^1^H MRS and reduced TUNEL-positive cells (estimated mean 47.1 cells/mm^2^ in HT+M versus 123.8 cells/mm^2^ in HT). An important finding was that in HT+V, ethanol, used to improve melatonin solubility, was associated with partial protection, based on aEEG recovery from 31 h and reduced TUNEL- positive cells (97.5 cell/mm^2^, midway between HT and HT+M). Increased oligodendrocytes were seen in both HT+V and HT+M versus HT, suggesting ethanol drove oligodendrocyte protection. Melatonin levels reached the putative therapeutic range (15–30 mg/l) at 2 h and 26 h after HI and remained within range 12–18 h after each infusion.

Melatonin was safe and did not lead to any physiological change at this high dose with a rapid infusion rate. Indeed, more dopamine and dobutamine were required to maintain the mean blood pressure in HT and HT+V versus HT+M, but there was no difference in fluid bolus requirements. Our previous piglet studies did not observe any effect of melatonin on blood pressure at doses up to 30 mg/kg^13,14^. Melatonin therapeutic levels (15–30 mg/l) were reached at 2 h using this rapid infusion started 1 h after HI. It is feasible that in babies with NE, resuscitation would be completed and venous access in place by 1 h, enabling the start of melatonin infusion. Oral melatonin has been used in NE babies undergoing HT and is absorbed^33^,^34^, however for rapid achievement of therapeutic levels and optimal protection, intravenous administration is more reliable. In the piglet model we have observed that brain protection is dependent on the time after HI that therapeutic levels are achieved^14^. This fits with melatonin’s diverse anti-oxidative effects which act upstream in the neurotoxic cascade to prevent free radical-induced oxidative damage to the electron transport chain and mitochondrial DNA^35^. The newborn infant is at heightened risk for free radical production and injury from oxidative stress. Melatonin and its metabolites work as free radical scavengers, by enhancing anti-oxidant enzyme expression and activity^36^. Further downstream, melatonin maintains mitochondrial energy production by increasing complex I and IV electron transport chain activity^37^. Melatonin subsequently prevents apoptosis by preventing nitro-oxidative damage to membrane lipids and inhibiting pro-apoptotic proteins such as BAX; these actions prevent cytochrome c leakage and propagation of intrinsic apoptotic cascades.

aEEG is used to monitor babies undergoing HT and the aEEG background voltage and rate of recovery after HI predict outcome^28^,^38^. In our study, mean aEEG scores recovered more quickly in HT+M versus HT from 19 h, but surprisingly, we saw improvement in HT+V versus HT from 30 h, corresponding to partial protection also seen with TUNEL-positive cells with HT+V.

MRS biomarkers predict 2-year neurodevelopmental outcome in NE infants^23^,^24^. Higher NTP and PCr on ^31^P MRS is associated with better 2-year outcome in clinical studies^26^; we saw higher ATP and PCr in HT+M versus HT at 48 h. ^1^H MRS Lac/NAA is a validated translational biomarker; high levels of thalamic Lac/NAA on MRS in NE babies predict poor 2-year outcomes^23^,^24^. We saw lower Lac/NAA on WM MRS with HT+M at 24 and 48 h and in grey matter at 48 h versus HT.

We saw an overall reduced number of TUNEL-positive cells in HT+M versus HT+V and HT. On regional assessment, most protection was observed in IC and pvWM. The high level of protection in these regions may relate to their increased vulnerability to free radical attack, localised severe injury and the early achievement of therapeutic melatonin levels. Although excitotoxic mediated injury may affect all neuronal cells, the myelin-producing oligodendrocytes are particularly vulnerable to injury. We observed that ethanol is likely to have particularly driven the oligodendrocyte protection in IC and pvWM. Other regions with lower levels of injury, such as the hippocampus, putamen and thalamus, also showed preserved oligodendrocytes with ethanol vehicle, but the effect was smaller. Here we did not measure changes in immature or mature oligodendrocytes or myelination. As melatonin is highly lipophyllic and easily penetrates brain and organelles, protection is unlikely to be due to vascular factors^39^. Protection of IC and pvWM with melatonin-augmented cooling as seen here would lead to improvements in cognitive and language outcomes with preservation of brain growth.

Compared to our recent study where therapeutic levels were achieved at 8 h after HI^14^, earlier administration of melatonin and therapeutic levels by 2 h in this current study provided enhanced protection. Here, pvWM and IC protection were similar to that achieved with melatonin administered at 10 mins after HI previously where we also saw protection in grey matter regions^13^. Such widespread protection may have been related to the overall more severe injury, expanding the potential for brain protection with melatonin-augmented cooling. Taken together, it is likely melatonin-augmented cooling would be effective in severe injuries and protect both white and grey matter.

There was clear partial protection seen with HT+V (ethanol) whereby mean TUNEL-positive cells/mm^2^ (97.5) were midway between HT (123.8) and HT+M (47.1). There was preservation of oligodendrocytes with HT+V mainly in IC and pvWM and ethanol is likely to have driven protection seen with HT+M in these regions. Ethanol vehicle protection was maximal in IC where injury was most severe. Ethanol has been used as a vehicle for several melatonin neuroprotection pre-clinical and clinical studies, including the piglet^13^, fetal sheep^15,18,19^ and human baby^16^. Ethanol modulates GABA and increases GABAergic neurotransmission^40^, this being inhibitory in adults but excitatory in immature brain^41^. Neuroprotection with acute low-dose ethanol given up to 4 h after middle cerebral artery occlusion is described in adult stroke studies with 1.0–1.5 g/kg ethanol producing blood levels of 80–100 g/dl^22^. In adolescent but not adult rats, high ethanol doses to model binge drinking, upregulated Toll like receptor signalling (TLRS) and increased proinflammatory cytokines^42^, supporting the concept that the immature brain is more vulnerable to ethanol’s toxic effects. This increased vulnerability of the developing brain to ethanol, related to inhibition of cortical activity^43^, mandates ethanol-free medicines for newborn infants^44^.

Therefore, it is likely that ethanol influenced protection in one^13^ but not another^14^ previous piglet study and in fetal sheep neuroprotection studies^15^,^17^–^19^,^45^. Only one fetal sheep study^15^ had an ethanol-only control group where ethanol exposure was associated with improved neuronal survival in striatum but reduced survival in hippocampus. In this study, ethanol was associated with suppression of cell proliferation and increased microglial activation, suggesting it is imperative to study melatonin with safe excipients in future studies.

We saw no effect of HT+M versus HT on brain microglial activation^13,14^. It is possible that, despite no visible change, gene expression was altered^13^. There was no effect of HT+M on GFAP. There were clear increases in CC3 in HT+M versus HT+V and HT. Such CC3 increases were evident throughout the brain; this may reflect caspase’s non-apoptotic functions, promoting microglial and lymphocyte function, cell differentiation and autophagy^46^–^48^. Increased CC3 expression, not linked the cell death, has been observed in the contralateral hemisphere in a unilateral hypoperfusion P7-rat model^49^. We have previously observed discrepancies between TUNEL-positive cell death and CC3 in our piglet model^32^. The use of male piglets may partly explain these data; cell death is dimorphic, and in males, apoptosis occurs via caspase-independent pathways^50^,^51^. It is likely that the observed TUNEL-positive cell death occurred independently of caspase 3^47^, such as necrosis, necroptosis, and non-caspase mediated apoptosis^52^. CC3 is therefore a poor cell death/apoptotic marker in this male piglet model.

There are limitations to this study. The inclusion of male piglets only was to minimize variability; inclusion of both sexes is necessary for future development of the model. Termination at 48 h, may not have allowed sufficient time for evolution of apoptotic cell death. Importantly, the rectal temperature was higher in HI versus HI+M (38.9 °C versus 38.2 °C). This is a potential confounder; cooling trials have shown that death/disability is increased 3.6–4.0 fold for each 1 °C increase in temperature in controls^53^. Duration of temperature rise influences outcome; the difference in our study was only 1 h; over the next 48 h, there was no difference. Nevertheless, temperature rise after HI, exacerbates injury^54^,^55^; and this could have worsened brain injury in HT. The blood pH was more alkalotic and PaCO_2_ lower at 24 h after HI in HT+M and HT+V versus HT. High variability in CO_2_, in particular hypocapnia^56^,^57^, is associated with NE adverse outcome and this could exacerbate HT+M brain injury. The contribution of phenobarbitone to brain injury is unclear^58^,^59^.

In the search for safe and effective therapies to improve outcomes in NE babies, it will be important to tailor therapies based on sex, inflammatory state and injury severity^60^. Melatonin may be an intervention which targets severe injury if started early enough. With its potent anti-oxidative effects at high dose, low side effect profile and lack of cold-chain storage requirement, melatonin has potential to improve NE outcomes. Phase I safety studies and careful incremental dose studies are needed prior to RCTs. There is a growing interest in the combination of agents with different neuroprotection profiles; melatonin with erythropoietin in preterm brain injury shows promise^61^.

In conclusion, 18 mg/kg melatonin 1 h after HI, combined with cooling, reduced brain injury based on faster aEEG recovery 19 h after HI, improved brain energy metabolism on ^31^P and ^1^H MRS over 48 h and reduced TUNEL positive cells with particular protection in most severely damaged regions, IC and pvWM. The vehicle, ethanol, used to improve melatonin solubility, led to partial protection based on aEEG recovery 31 h after HI, reduced TUNEL positive cells and increased oligodendrocytes in IC and pvWM versus HT. Melatonin is a promising and safe neuroprotective agent which augments HT if target therapeutic levels are achieved ~2 h after HI. It is imperative to study melatonin with ethanol-free excipients in future pre-clinical and clinical neuroprotection studies.

## Supplementary information

Supplementary Information.
