# Supplementary material for: High-Dose Melatonin and Ethanol Excipient Combined with Therapeutic Hypothermia in a Newborn Piglet Asphyxia Model
Source: Sci Rep. 2020 Mar 3;10:3898. doi: 10.1038/s41598-020-60858-x (PMC7054316; doi:10.1038/s41598-020-60858-x)
Supplement: Supplementary file 1 — Supplementary Information. [file 41598_2020_60858_MOESM1_ESM.docx]

**Supplementary file**

**Sample size calculation**

Our primary outcome was thalamic and white matter Lac/NAA. Previous studies have indicated that the change in Lac/NAA during 48h after HI varied between HT and HT plus melatonin by 0.5U with a standard deviation of 0.3U (log scale). Assuming a similar effect magnitude with melatonin and similar variability at 48h and with 5% significance and 80% power, at least 7 subjects are needed in each group.

**Surgical Preparation**

Anesthesia was induced by 4% v/v isoflurane through a facemask to facilitate tracheostomy and intubation. Throughout the surgery, isoflurane was maintained at 2.5-3% guided by peripheral oxygen saturation monitoring (Nonin Medical, Plymouth, MN, USA) and the animal’s response to stimulation. Following tracheostomy, a suitable size endotracheal tube (Smiths Medical, Ashford, Kent, UK) was fixed and the piglet was mechanically ventilated (SLE 2000 infant ventilator, Surrey, UK). Ventilator settings were adjusted to maintain partial pressure of oxygen (PaO_2_) at 8–13kPa and carbon dioxide (PaCO_2_) at 4.5–6.5kPa, allowing for temperature correction of the arterial blood sample.

After the airway was secured, both common carotid arteries were surgically isolated at the level of the fourth cervical vertebra and a vascular occluder (OC2A, In Vivo Metric, Healdsburg, CA, USA) was placed on each side. After completion of surgery, inspired isoflurane concentration was maintained at 2% v/v.

A 4 French double lumen umbilical venous catheter (Vygon, Swindon, UK) was inserted for infusion of maintenance fluids (10% dextrose, 60 ml/kg/day, reduced to 40ml/kg/day post insult), fentanyl (3μg/kg/h) and antibiotics (benzylpenicillin 50 mg/kg every 12h and gentamicin 2.5 mg/kg every 24h). A 2.5 French umbilical arterial catheter (Vygon) was inserted for continuous monitoring of heart rate and mean arterial blood pressure (MABP), and intermittent blood sampling was used to measure PaO_2_, PaCO_2_, pH, electrolytes, glucose and lactate (Abbot Laboratories, UK). Arterial lines were maintained by infusing 0.9% saline solution (0.3mL/h) with 1 IU/mL heparin sodium.

All piglets received continuous physiological monitoring (SA instruments). To maintain the MABP >40mmHg, bolus infusions of 0.9% saline (Baxter; 10 ml/kg), dopamine (5–20μg/kg/min), dobutamine (5–20μg/kg/min), noradrenaline (0.1-1μg/kg/min) and adrenaline (0.1–1.5μg/kg/min) were used as required by a NICU trained clinician. Hyperkalemia (>7.0mmol/l) was treated with 4μg/kg salbutamol (10μg/ml) over 10 min. Rectal temperature was maintained at 33.5^o^C from 1-13h using a cooling water mattress (Tecotherm); rewarming occurred at 0.5^o^C/h. For the remainder of the study rectal temperature was maintained in the normothermic range (38.0-39.0^o^C) using a radiant warmer during surgery and subsequently a heating water mattress (Tecotherm).

**aEEG scoring**

Scoring was performed by two clinicians blinded to the treatment allocation (IL, KM). Hourly aEEG scores were averaged in 6h time epochs and mean differences analyzed for differences between study groups. Particular note was taken of aEEG background activity over the 1h after resuscitation; recovery to discontinuous normal voltage or continuous normal voltage triggered exclusion, as the insult was considered too mild (based on clinical practice in the UK TOBY xenon study for NE where normal aEEG background voltage excluded a baby from entering neuroprotection trial protocols) (Azzopardi et al., 2015).

**Magnetic Resonance Spectroscopy Analysis**

Nucleotide triphosphate (NTP) peaks were fitted as a doublet (α,γ) and triplet (β) structure with no assumption of the relative multiplet sizes. NTP is predominantly composed of adenosine triphosphate (ATP) and therefore changes in this signal reflect levels of this high-energy phosphate molecule. Measurements of inorganic phosphate (Pi), phosphocreatine (PCr), exchangeable phosphate pool (epp = *Pi + PCr + 2γ-NTP + β-NTP*) were acquired over the whole brain and peak area ratios calculated (Pi/epp, PCr/epp, and NTP/epp). ^1^H MRS spectra were acquired using body coil transmit and with a separate 6.5 x 5.5 cm elliptical receive surface coil tuned to the ^1^H resonance frequency.

**Melatonin assay**

Blood samples were collected in lithium/heparin tubes and centrifuged immediately after collection. Plasma was separated and then stored at -20°C before analysis. When piglets were euthanized, in most cases a terminal cerebrospinal fluid (CSF) sample was collected and melatonin levels analyzed. Plasma melatonin levels were measured by Stockgrand Ltd following a modification of Fraser (Fraser, Cowen, Franklin, & Lewy, 1983). Samples were subjected to chloroform extraction, solvent was removed by evaporation under oxygen-free nitrogen. Extracted melatonin was resuspended in assay buffer and further diluted to give concentrations within the standard curve range of 0 – 500pg/ml. Pig plasma quality control samples and spiked pig plasma were included in each assay. Limit of detection of the assay was 3pg/ml. Quality control values were 27.8 ± 3.4pg/ml coefficient of variation (CV) = 12.3%; 73.5 ± 7.9pg/ml CV = 10.7%; 118.1 ± 12.7pg/ml CV = 10.7%; 214.2 ± 27.6pg/ml CV = 12.9%; 137.9 ± 21.1ng/ml CV = 15.3%; 256.1 ± 38.9ng/ml CV = 15.2%; 550.0 ± 37.2ng/ml CV = 6.8%.

**Immunohistochemistry methodology**

To assess cell death and glial activation, sections were stained for nuclear DNA fragmentation using histochemistry with terminal deoxynucleotidyl transferase dUTP nick end labelling (TUNEL); the appearance of activated caspase 3 (CC3); glial fibrillary acidic protein (GFAP); and microglial ionised calcium-binding adaptor molecule (Iba1) immunoreactivity. Oligodendrocytes were stained with oligodendrocyte transcription factor (OLIG2). For each animal, 2 sections placed 5mm apart (bregma 00 and -2.0) were assessed for each stain.

For all histochemical and immunohistochemical stains, brain sections were dehydrated in xylene (3 × 10 min) and rehydrated in graded ethanol solutions (100–70%), followed by double-distilled water. For TUNEL, endogenous peroxidases were removed by pre-treating sections in 3% H_2_O_2_ in methanol, followed by a 15-min peptidase pre-digestion with 20 µg/ml proteinase K (Promega) at 65°C. Sections were then incubated at 37°C for 2 h with the TUNEL solution (Roche) containing biotinylated dUTP. TUNEL positive cells were counted in 3 fields (x40 magnification; area 0.066 mm^2^) and averaged per region (cells / mm^2^).

For activated caspase 3, Iba1 and OLIG2; pre-treatment with Ventana CC1 (950-124), equivalent to EDTA buffer was used. For GFAP, Protease 1 (0.38 mg/mL alkaline protease enzyme activity) was used. Primary antibody incubation was performed with primary rabbit antibody against activated Caspase 3 (1:100, Cell Signalling 9661L) for 32min, Iba1 (1:250, WAKO 019-19741) for 4hrs, GFAP (1:1000, DAKO Z0334) for 32min and OLIG2 (1:100, Millipore AB9610) for 4h. Incubation with a secondary swine anti-rabbit immunoglobulin (DAKO E0343) was performed for a duration of 44min in activated caspase 3; 1hr for Iba1 and OLIG2; and for 32min in GFAP staining.

The biotin residues were detected with the avidin-biotinylated horseradish peroxidase complex (ABC, Vector Laboratories) and visualized with diaminobenzidine/H_2_O_2_ (Sigma), with CoCl_2_ and NiCl_2_ included to intensify TUNEL histochemistry. The sections were dehydrated in graded alcohol and xylene and mounted with Depex (VWR), or alternatively, mounted with Vectashield + 4',6-diamidino-2-phenylindole (DAPI) aqueous mounting media (Vector Labs), to facilitate total cell number counts during analysis of Iba1 and activated caspase 3.

Iba1 positive microglial cells were assigned a ramification index based on their body and branch density using a 0.049 x 0.049mm square grid (x40 magnification) placed in three fields for each brain region. The microglial ramification index was calculated as *B^2^/C*, where B represents the average number of branches crossing the 3 horizontal and 3 vertical 0.049mm gridlines and C represents the number of cell bodies within the grid. Activated caspase 3 immunoreactive cells were counted in 3 fields (x20 magnification; area 0.164 mm^2^) and averaged per region (counts / mm^2^). OLIG2 positive cells were counted in 3 fields (x40 magnification; area 0.066 mm^2^) and averaged per brain region (cells / mm^2^). To quantify the total intensity of the GFAP staining in the tissue, we used optical luminosity values as a well‐established technique (Möller et al., 1996). GFAP is expressed not just by the bodies, but also in the processes of astroglial cells. The images were captured with a Sony AVT‐Horn 3CCD colour video camera (24bit RGB, 760 × 570 pixel resolution) in three different optical fields in the eight brain regions, as well as the surrounding glass at ×20 magnification. We used Optimas 6.5 software to obtain the mean and standard deviation (SD) for optical luminosity values (OLV). SD was subtracted from the mean for each image and the resulting value was subtracted from the values obtained for the surrounding glass.

**Statistical Analysis**

Statistical analysis was performed using Prism version 6.0 for Mac, GraphPad Software, La Jolla California USA. Parametric data was analyzed using a student t-test and non-parametric data with Mann-Whitney U test. MRS, aEEG and immunohistochemistry data were analyzed using an ANOVA of the least mean squared difference. Histology data was log_10_ transformed to normalize distribution for parametric statistical analysis.

**References**

Azzopardi, D., Robertson, N., Bainbridge, A., Cady, E., Charles-Edwards, G., Deierl, A., . . . Edwards, A. (2015). Moderate hypothermia within 6 h of birth plus inhaled xenon versus moderate hypothermia alone after birth asphyxia (TOBY-Xe): a proof-of-concept, open-label, randomised controlled trial. *Lancet Neurol, S1474-4422(15)00347-6*.

Fraser, S., Cowen, P., Franklin, M., & Lewy, A. (1983). Direct radioimmunoassay and gas chromatography-mass spectrometry compared for determination of melatonin in plasma. *Clin Chem, 29*(9), 1703-1704.

Möller, J., Klein, M., Haas, S., Jones, L., Kreutzberg, G., & Raivich, G. (1996). Regulation of thrombospondin in the regenerating mouse facial motor nucleus. *Glia, 17*(2), 121-132.
